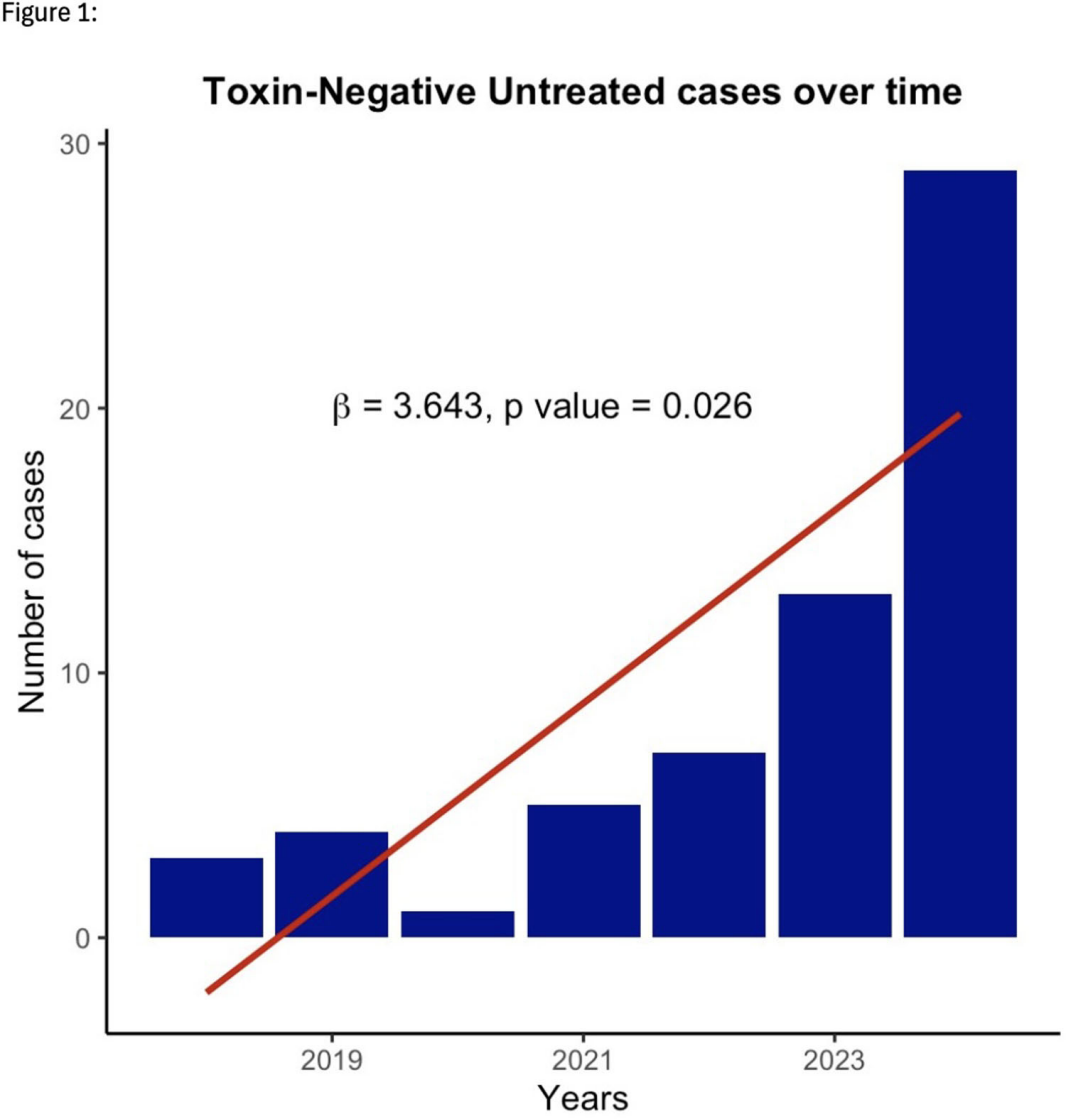# 139 Healthcare-Associated Bloodstream Infections in an Adult ICU in Vietnam: Findings From a Multi - Year Infection Prevention Surveillance

**DOI:** 10.1017/ash.2026.10547

**Published:** 2026-06-23

**Authors:** Christian Wang, Fabian Patauner, Olivia Howland, Jennele Baul, Amy Pulikeyil, Keith Kaye, John Mills

**Affiliations:** 1 Rutgers Robert Wood Johnson Medical School; 2 Princeton Infectious Diseases Associates

## Abstract

**Background:** Patients with NAAT positive/toxin EIA-negative C. difficile infections (TNCDI) represent a spectrum from fulminant infection to asymptomatic colonization. We assessed differences in patient and provider factors associated with TNCDI treatment. **Methods:** A retrospective review was performed of all hospitalized patients ≥ 18 years with TNCDI from Jan 1st 2018 to Dec 31st 2024. Patients who received anti-CDI treatment were compared to those who did not using chi squared and Mann-Whitney U test. Variables with P≤0.2 were considered for inclusion in the final multivariable model. **Results:** We identified 550 patients with TNCDI; 488 (88.7%) received treatment while 62 (11.3%) did not (Table). The annual number of cases of untreated TNCDI significantly increased from 2018 to 2024 (p=0.026) (Figure). There were no significant differences in age, race, BMI, or provider services between the two groups. A higher proportion of untreated TNCDI occurred among women and in community-acquired cases. In multivariate analysis, community-acquired diagnosis of infection (within 72 hours of admission) was the only significant predictor of treatment (OR for treatment 0.50, p=0.03) (Table). In unadjusted analysis, treated TNCDI patients had higher LOS after diagnosis and mortality compared to untreated patients. **Conclusion:** The vast majority of TNCDI received anti-CDI treatment, though the proportion of untreated TNCDI cases increased significantly over time, likely reflecting antibiotic stewardship-based improvements. Patient and provider factors were similar between treated and untreated TNCDI groups, although small sample size of untreated TNCDI patients limited power. Treated TNCDI was associated with significantly longer LOS and mortality compared to untreated TNCDI.

**Figure:**
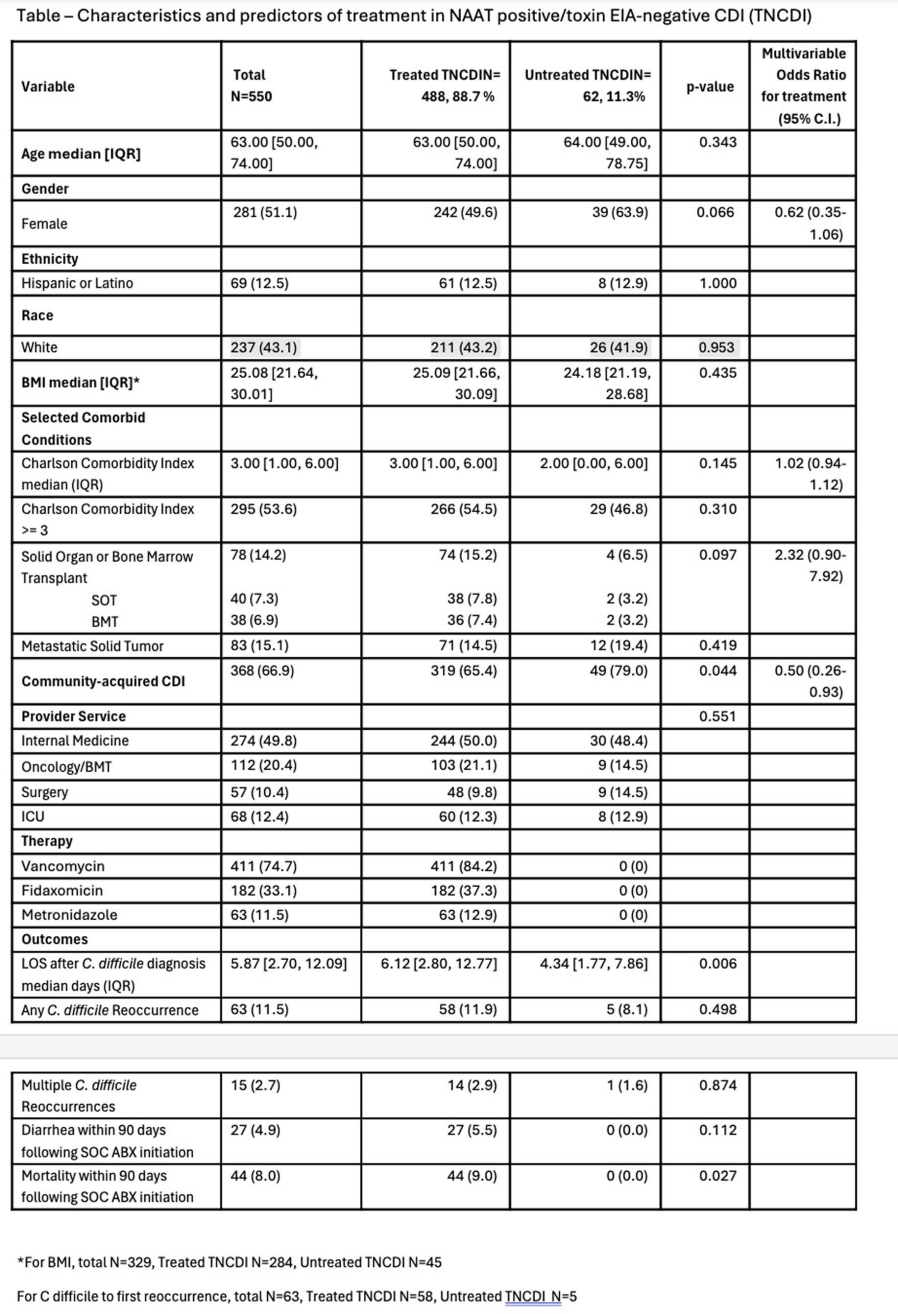

**Figure:**